# Suicidal Ideation and Predictors of Psychological Distress during the COVID-19 Pandemic in Eswatini: A Population-Based Household Telephone Survey

**DOI:** 10.3390/ijerph18136700

**Published:** 2021-06-22

**Authors:** Mduduzi Colani Shongwe, Song-Lih Huang

**Affiliations:** 1 Institute of Public Health, College of Medicine, National Yang Ming Chiao Tung University, Taipei 112, Taiwan; mduyaye@gmail.com; 2 International Health Program, College of Medicine, National Yang Ming Chiao Tung University, Taipei 112, Taiwan; 3 Department of Midwifery Science, Faculty of Health Sciences, University of Eswatini, Mbabane H100, Eswatini

**Keywords:** anxiety, coronavirus, coronavirus disease, COVID-19, mental health, SARS-CoV-2, psychological distress

## Abstract

The unpredictability of the COVID-19 pandemic can induce psychological distress in individuals. We investigated perceived stressors, prevalence of psychological distress and suicidal ideation, and predictors of psychological distress among adults during the COVID-19 pandemic in Eswatini. This study was a cross-sectional, population-based household telephone survey of 993 conveniently sampled adults (18+ years) from all the four administrative regions of Eswatini. Data were collected between 9 June and 18 July 2020 during the first wave of the COVID-19 pandemic, when the country was under a partial lockdown. COVID-19-related psychological distress was assessed using the Kessler 6-item Psychological Distress Scale (K6). We performed weighted modified Poisson regression analyses to identify significant predictors of moderate/severe psychological distress (K6 scores: ≥5). The weighted prevalences of moderate (K6 scores: 5–12) and severe psychological distress (K6 scores: ≥13) were 41.7% and 5.4%, respectively. Participants reported several perceived COVID-19-related stressors, including worries and fears of the contagion-specific death, serious need for food and money, and concerns about loss of income or business. The weighted prevalence of suicidal ideation was 1.5%. Statistically significant predictors of increased risk for moderate/severe psychological distress included living in the Hhohho and Manzini regions; feeling not well informed about COVID-19; feeling lonely; having received COVID-19 food or financial relief from the government; feeling burdened by the lockdown; being married; and being youth (18–24 years). The results call for the government to urgently augment the provision of mental health services during the pandemic. Mental health practitioners and programs may use several stressors and risk factors identified in this study to inform interventions and government policies aimed at reducing psychological distress induced by the pandemic.

## 1. Introduction

The novel coronavirus disease (COVID-19), caused by severe acute respiratory syndrome coronavirus 2 (SARS-CoV-2), is an ongoing global health emergency. By 13 June 2021, there had been more than 175.3 million confirmed cases of COVID-19 globally, including more than 3.7 million deaths, of which more than 3.6 million of the global cases were from Africa, including 89,674 deaths [1]. In Eswatini, which is a small, landlocked, lower–middle-income country in Southern Africa with a population of about 1.1 million [2], the first case of COVID-19 was reported by the Ministry of Health (MoH) on 13 March 2020. By 13 June 2021, there had been 18,736 confirmed COVID-19 cases, including 676 deaths in the country [3], translating to a 3.6% case fatality rate–one of the highest in the African region and around the world [4]. Due to Eswatini’s proximity to South Africa (the country with the highest number of COVID-19 cases in Africa), Eswatini found itself one of the severely impacted countries in the region. The recent genetic sequencing of samples from confirmed cases in Eswatini revealed a prevalence of 88% of the SARS-CoV-2 20H/501Y.V2 or B.1.351 variant [5], first reported in South Africa in late 2020 [6].

COVID-19 has resulted in bereavement, isolation, loss of income, and fear, which are potential triggers of mental health conditions or may exacerbate existing ones. As a result of COVID-19, many people may face increased levels of alcohol and drug use, insomnia, and anxiety, while COVID-19 itself leads to neurological and mental complications, such as delirium, agitation, and stroke. People with pre-existing mental, neurological, or substance use disorders are also more vulnerable to SARS-CoV-2 infection and stand a higher risk of severe outcomes, including death [7]. In addition, the containment measures proposed by governments and public health experts (i.e., quarantine, isolation, social distancing, and mandatory lockdowns), the unpredictability and evolving nature of the pandemic, uncertainties regarding a cure, and widespread misinformation, especially on social media, can induce psychological distress among the general public [8,9]. 

The prevalence of psychological distress varies by country. A multicountry cross-sectional survey of 678 participants (predominantly from the USA, Pakistan, Canada, and the UK) found that 50.9% of participants showed traits of anxiety and 58.6% of participants exhibited depression [10], whereas another U.S. study found a prevalence of 70.4% moderate distress [11]. A recent narrative review also found that symptoms of anxiety and depression (16–28%) and self-reported stress (8%) were common psychological reactions to the COVID-19 pandemic [12]. In Nigeria, 51% of participants reported moderate anxiety, and 49% of participants had severe anxiety during the pandemic [13]. In a survey of 221 adults in South Africa, participants reported experiences of anxiety and 14.5% of participants were at risk of depression [14], whereas in Uganda and Zambia, a survey of 12,000 women revealed an increase in persistent stress, anxiety, and depression during the pandemic [15]. With regard to suicides, in Bangladesh, seven of eight suicide cases were attributed to economic issues related to COVID-19 [16] . In New Zealand, suicidal ideation was reported by 6% of participants, with 2% of participants reporting making plans for suicide, 2% of participants reporting suicide attempts, and under 10% of participants reporting experiencing some forms of family harm over the lockdown period [17]. However, in both countries, recent pre-pandemic actual rates of suicide are not readily available to make comparisons [17,18].

Factors known to be associated with increased psychological problems during the COVID-19 pandemic include higher perceived COVID-19 risk [14], being aged 30–59 years old, living with comorbidities [19], increased smoking, high levels of fear, change of employment status, and providing care to known or suspected COVID-19 cases [20]. Others include being aged 18–24 or 25–34 years old, being female, being a student, having physical symptoms and poor self-rated health status [21], living with young children, being employed before the pandemic [22], living in rural areas, having a lower socioeconomic status [23], and marital status [10,24]. On the contrary, having up-to-date and accurate health information and taking precautionary measures have been found to be protective against stress, anxiety, and depression [21]. However, factors associated with psychological distress in Southern Africa during the ongoing pandemic have not been widely investigated [25].

Even before the COVID-19 pandemic, Africa had one of the lowest mental health public expenditure rates [15], indicating the poor prioritization of mental health in the region. In a WHO global survey examining the devastating impact of COVID-19 on access to mental health services, 27 of 28 African nations that took part in the survey reported having included mental health in their COVID-19 response plans. However, 37% of them reported that their plans were only partially funded, whereas another 37% of them reported having no funds at all [15]. In response to the pandemic, some African governments set up counseling helplines and increased training in basic psychosocial skills for key health responders [26]; however, this did not happen in Eswatini. 

Eswatini is faced with multiple socio-economic challenges, including the highest HIV prevalence (at 27%) among adults (15 years and older) in the world [27], the second highest double burden of HIV–TB co-infection (at 70%) in the world [28], and high burdens of diabetes mellitus (14.2%) and hypertension (24.5%) [29], which are all known risk factors for COVID-19 infection [30,31]. Despite agriculture being the main driver of the economy and subsistence farming being the major source of food for many families, about 59% of the population live below the poverty line [32], whereas the unemployment rate stands at 28.2% among the general population [2]. The aforementioned country situation complicates the potential socio-economic and psychological impact of the pandemic on the populace and places the country in a uniquely precarious position to be severely impacted by the COVID-19 pandemic than other African countries.

Unfortunately, the pandemic also caught Eswatini without a mental health policy [33], and mental health services remain largely cantered at the only psychiatric hospital in the country [34], further hindering any mitigation efforts against the mental health impact of the pandemic. This also occurs against a backdrop of an already resourced-strained health care system marked by chronic staff shortages, limited national budget allocations, shortage of drugs, overcrowding in health facilities, and inadequate high care facilities and equipment [34,35]. 

Preliminary reports from the Eswatini Royal Police indicate that during the early phase of the partial lockdown (i.e., between 27 March and 19 April 2020), 299 sexual and gender-based violence cases had been reported, 53 of which were rape cases, and the rest were domestic violence cases [35]. Recent pre-pandemic data to enable direct comparison during the same period are not readily available. However, a 2017 nationally representative survey found that 5.2% and 0.2% of adult women reported physical and sexual violence in the 12 months prior to the survey, respectively [27]. To the best of our knowledge, no study has investigated the mental health of the Eswatini population during the pandemic. It is against this backdrop that we conducted this study due to the following reasons: (1) to determine the perceived stressors (i.e., fears, concerns, and critical or serious needs) related to COVID-19 and the lockdown, (2) to describe the prevalence of psychological distress and suicidal ideation during the pandemic, and (3) to determine the predictors of moderate/severe psychological distress among the general public during the COVID-19 pandemic in Eswatini.

## 2. Materials and Methods

### 2.1. Study Design and Setting

This study was a cross-sectional, population-based household telephone survey conducted in all four administrative regions of Eswatini. More than three-quarters (76.2%) of Eswatini’s population live in rural areas, with about 90% identify themselves as Christians, and the country has a predominantly youthful population with a median age of 21.7 years [2]. 

### 2.2. Study Population and Sampling Procedure

Our source population was adults living in households with a telephone line provided by the Eswatini Posts and Telecommunications Corporation (EPTC), the sole landline telephone service provider in the country, with approximately 50,000 subscribers (including commercial subscribers), representing approximately 4.5% of the country’s population. We used a single-stage stratified random sampling strategy to select households from a list of all residential landline telephone numbers abstracted from the hard copy of the 2020 EPTC Telecommunications Directory. The regional codes were used to stratify the telephone numbers by region, which were reconfirmed by participants during the interviews. To create our sampling frame, we first entered all residential telephone numbers manually into four Microsoft Excel spreadsheets, and random numbers were generated for sampling until the required sample size was reached per region. Households with faulty lines or no response after two separate attempts were replaced with the next telephone number on the random list. Within each household, we used convenience sampling to select one adult family member (≥18 years), irrespective of their gender, who was available for an interview, but first preference was given to household heads and all participants had to be Swati. We excluded those with hearing problems. 

### 2.3. Sample Size Determination

**Figure 1:**
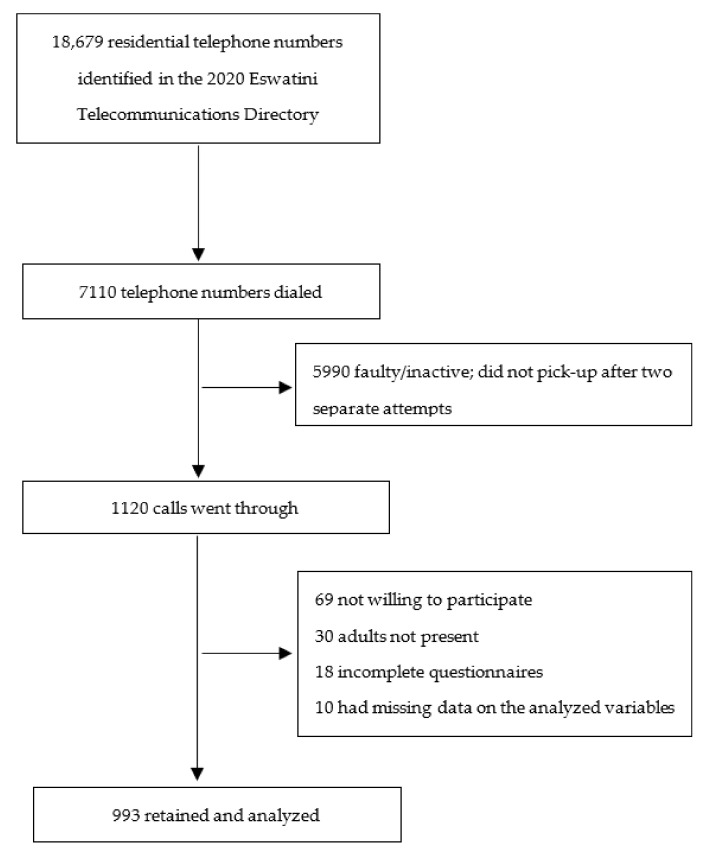
A schematic view of the sampling flow.

We employed probability proportional to size (PPS) sampling by region based on population estimates for the most recent (2017) Eswatini Population and Housing Census [2]. Since the aim of the study was to generalize the findings for the general adult population of Eswatini, a large sample size (*N* = 1000) was targeted [36]. Thus, data collection was terminated, when 1003 participants were interviewed, of which 10 were excluded for missing data on the studied variables, resulting in a final sample of 993. Figure 1 shows the sampling flow.

### 2.4. Data Collection

Data collection commenced on 9 June 2020 and ended on 18 July 2020. During this period, the country was still in the first wave of the pandemic and was under a partial lockdown whereby COVID-19 cases and deaths were on the rise. The telephone interviews were conducted in the language (English or SiSwati) preferred by the participant, as English is the official language of doing business in Eswatini and is the language of instruction from kindergarten up to the highest level in tertiary education. The English language version of the questionnaire was forward-translated into Siswati by a native bilingual Swati and thereafter submitted to another bilingual native to cross-check if the translated version reflected the original scale, of which it was found to be the case. In addition, two native experts independently reviewed the translated version and concurred that it reflected the original English version of the scale. Prior to the main data collection, the questionnaire was pretested among 10 participants, and there were no modifications that ensued from the pretest as the instrument was found to work well. On average, each interview lasted for about 15 min, including time for informed consent procedures. Seven data collectors conducted the interviews, all of whom had prior pre-service training on research methods, and we further provided them with a two-hour virtual refresher training on research ethics, study procedures, and interviewing skills.

### 2.5. Measures

#### 2.5.1. Outcome Variables

Our primary outcome, psychological distress, was measured using the well-validated Kessler 6-item Psychological Distress Scale (K6) [37,38]. The K6 items assess the frequency of nonspecific psychological distress within a particular reference period [39]. In this study, we used the interviewer-administered version of the scale where each question was phrased with a specific reference to the pandemic, e.g., “Since the COVID-19 pandemic started, about how often did you feel (hopeless)—all of the time, most of the time, some of the time, a little of the time, or none of the time since the pandemic started in Eswatini?” Thus, each item was measured on a 5-point Likert scale ranging from 0 (“none of the time”) to 4 (“All of the time”). Scores on the scale were summed up to create a composite score ranging from 0 to 24, where values of 0–4 indicated none-to-low distress, values of 5–12 indicated moderate distress, and values of ≥13 indicated severe psychological distress [39]. The K6 has a well-established cross-cultural validity [40], and in this study, its Cronbach’s alpha was 0.79.

In this study, we dichotomized the outcome variable to 0 representing “none/low distress” for K6 scores of 0–4 and 1 representing “moderate/severe distress” for K6 scores of ≥5, similar to other studies [11,41,42,43,44]. We used the sub-threshold (K6 scores: ≥5) for two reasons: first, we considered a score of ≥5 as clinically significant [39,41,42]. Prochaska et al. [39] noted that, when used at the conventional cut-off points of ≥13, the K6 fails to capture individuals struggling with more moderate mental distress that nonetheless warrants mental health intervention, because mental illness manifests in different symptoms in different cultural groups while some ethnic groups may be less forthcoming in reporting psychological symptoms, such as in Southern Africa [39,45]. Second, using the cut-off points of ≥5 was a statistical consideration [42], after noting that there were few participants classified as severely distressed in this study (see Section 2.3).

Our secondary outcome of interest was suicidal ideation [46,47]. We asked participants to report whether they had thought of killing themselves due to the pandemic. 

#### 2.5.2. Explanatory Variables 

All explanatory variables were chosen based on our review of previous literature on psychological distress [20,22,48,49]. These include age; sex; residence (urban/rural); region; marital status; the highest level of education attended; subjective socio-economic status (“Comparing your household to other households in your community, how do you consider your socio-economic status?”); knowing someone diagnosed with COVID-19; self or family member losing job/business due to the pandemic; having received COVID-19 financial or food relief from the government; perceived ability to avoid contracting coronavirus; perceived probability to contract coronavirus; and perceived severity if sick with COVID-19. Participants were also asked to report on their sources of stress or perceived stressors related to the pandemic [50], including their main fears, serious or critical needs, and experience of any form of abuse during the lockdown. We also asked participants if they ever felt lonely and worried such that they could not sleep at night during the pandemic.

### 2.6. Statistical Analysis

Data were analyzed in Stata 15 [51]. We used the complete case method to handle missing data and employed model-based weighting proposed by Valliant and Dever [52] to construct super population weights for a nonprobability survey using the svycal regress command. As a result, all weighted analyses were post-stratified by age, sex, residence, and region based on population totals (controls) from the 2017 Eswatini Population and Housing Census [2]. Thus, weighted frequencies, proportions, and means were computed to describe sample characteristics and main study variables. We performed weighted modified Poisson regression [53,54] (i.e., with a robust error variance obtainable automatically through the svy command) to directly estimate prevalence ratios for moderate/severe psychological distress (K6 scores: ≥5), which were equivalent to risk ratios (RRs), similar to the analytic methods used by Scheim, et al. [55]. However, since some studies use the cut-off points of ≥13, we conducted a sensitivity analysis using ordinal logistic regression with the three-category measure of psychological distress (none/low distress (K6 scores: 0–4): 0; moderate distress (K6 scores: 5–12): 1; and severe distress (K6 score: ≥13): 2) to illustrate the consistency of our results at K6 scores of ≥5 and ≥13. We used the likelihood ratio test to assess if the proportionality of odds assumption was not violated.

In multivariate analyses, we only retained statistically significant predictors in the final model, except for the variable “sex”, due to its known theoretical importance in predicting mental illness in adults [56]. However, we could not perform weighted inferential statistical analyses for our secondary outcome (suicidal ideation), e.g., performing bivariate associations or fitting crude logistic regression models, because sample size requirements for inferential tests were not met since few participants reported suicidal ideation in this study. Nonetheless, we still performed unweighted Fisher’s exact tests which did not yield any statistically significant associations between the potential predictors of suicidal ideation, except for one variable. Otherwise, for all the other variables, we only reported descriptive crosstabulations. All inferential statistical tests were deemed statistically significant if the *p*-value was ≤0.05 (two-tailed).

### 2.7. Ethical Approval

The study was conducted according to the guidelines of the Declaration of Helsinki and was approved by the Eswatini Health and Human Research Review on 19 May 2020 (protocol reference number: SHR251/2020). Since this study involved a non-face-to-face data collection method (telephone interviews), a waiver of written consent was granted. We obtained verbal informed consent before each interview. All other research ethics principles (e.g., autonomy, confidentiality, privacy, anonymity, and data protection) inherent in research involving humans were ensured in this study.

## 3. Results

### 3.1. Sample Characteristics 

Table 1 shows the unweighted and weighted percentages to reflect the likely distribution of the background characteristics of the Eswatini population. After applying sample weights, the sample was consistent with the characteristics of the target adult (15+ years) population in Eswatini in terms of age, gender, residence, and region. In the weighted analysis, more than a third (36.2%) of the participants had (either themselves or a family member) lost a job or means of doing business due to the pandemic, 11.4% had received food or financial relief from the government, and 64.9% felt well informed about COVID-19. About 44% of the population reported difficulty in avoiding contracting the virus, whereas nearly half of the population (47.8%) thought their probability of contracting COVID-19 was either high or very high, and a third of the population (33.4%) thought they could be severely or very severely ill if they were to contract the disease.

### 3.2. Prevalence of Psychological Distress and Suicidal Ideation

The weighted prevalences of moderate and severe psychological distress were 41.7% (95% confidence interval (CI): 37.7%, 45.8%) and 5.4% (95% CI: 3.9%, 7.5%), respectively. Thus, overall, nearly half of the participants (47.2%; 95% CI: 43.1%, 51.3%) were classified as having moderate/severe psychological distress (K6 score: ≥5), whereas 1.5% of the participants (95% CI: 0.8%, 2.7%) reported thoughts of committing suicide during the first wave of the pandemic in Eswatini (Table 1).

### 3.3. Distribution of Participants’ Background Characteristics by Suicidal Ideation

Table 2 shows the weighted distribution of background characteristics of the sample by suicidal ideation. Of the 17 participants who reported suicidal ideation, the majority (i.e., highest weighted proportion) were aged 25–59 years (56.9%), were female (67.3%), were single (74.7%), had a high school education (47.4%), were living in rural areas (60.6%), were from households self-classified as being in the middle socioeconomic status (74.5%), and were from the Hhohho region (49.8%). Moreover, the majority of those who had suicidal thoughts were those who felt well informed about COVID-19 (87.8%), who found it very easy or easy to avoid contracting COVID-19 (54.1%), who thought their probability of contracting COVID-19 was high or very high (50.3%), who were not sure if they would be seriously ill if they were to contract COVID-19 (73.4%), who never or rarely felt lonely (87.1%), as well as those who had none/low psychological distress (63.0%). In unweighted analyses using Fisher’s exact test, only the variable, “knowing someone with COVID-19” was significantly associated with suicidal ideation (*p* = 0.02; Table 2).

### 3.4. Perceived COVID-19-Related Stressors

In the weighted analysis, 23.9% of the participants stated that they were afraid of the contagion. About one-fifth (20.8%) of the participants felt burdened by the lockdown, whereas 15% of the participants were worried about the risk of contracting the virus, and 21.4% of the participants said they sometimes/most of the time/always felt lonely (and worried such that they could not sleep at night due to the pandemic (22.4%). More than half of the participants (53.1%) feared dying from COVID-19, 58.4% of the participants were in serious need of food during the lockdown, and 15.7% of the participants were concerned about job losses, loss of income, or business (Table 3).

### 3.5. Predictors of Psychological Distress

#### 3.5.1. Bivariate Analysis Results

Table 4 shows the weighted crude and adjusted modified Poisson regression models predicting moderate/severe psychological distress among Eswatini adults during the COVID-19 pandemic. In bivariate analysis, those who had secondary and high school education had an increased risk of moderate/severe psychological distress than those who had attended tertiary education, with crude risk ratios (CRRs) of 1.58 (95% CI: 1.24, 2.02) and 1.29 (95% CI: 1.05, 1.60), respectively. Those who found it very difficult or difficult to avoid contracting COVID-19 had an increased risk of moderate/severe psychological distress (CRR: 1.41; 95% CI: 1.07, 1.85) than those who had moderate difficulty in doing so. Similarly, those who thought they could be very severely or severely ill if they were to contract the disease had an increased risk of moderate/severe psychological distress (CRR: 1.35; 95% CI: 1.08, 1.67) than those who thought they would be less severely ill or not severely ill (Table 4).

#### 3.5.2. Multivariate Analysis Results

In the multiple modified Poisson regression analysis (Table 4), holding other covariates constant in the model, the youth (18–24 years old vs. 25–59 years old) and those who were married or cohabiting vs. those who were single had increased risks of moderate/severe psychological distress, with adjusted risk ratios (ARRs) of 1.34 (95% CI: 1.05, 1.70) and 1.37 (95% CI: 1.09, 1.72), respectively. Compared to those living in the Lubombo region, those living in the Hhohho and Manzini regions had increased risks of moderate/severe psychological distress, with ARRs of 1.63 (95% CI: 1.24, 2.15) and 1.42 (95% CI: 1.07, 1.89), respectively, holding the other covariates constant in the model. Those who did not feel well informed about COVID-19 had a significantly increased risk of moderate/severe psychological distress with an ARR of 1.59 (95% CI: 1.32, 1.91) than those who felt well informed about the disease, holding the other covariates constant in the model. The risk of moderate/severe psychological distress was also significantly increased among those who received COVID-19 food or financial relief from the government (ARR: 1.35; 95% CI: 1.10, 1.65) than those who did not/were not sure. We also found increased moderate/severe psychological distress among those who felt burdened by the lockdown (ARR: 1.33; 95% CI: 1.11, 1.59) vs. those who were not, holding the other covariates constant in the model. Likewise, those who sometimes/most of the time/always felt lonely had an increased risk of moderate/severe psychological distress (ARR: 2.01; 95% CI: 1.23, 2.34) compared to those who rarely or never felt lonely during the pandemic, holding the other covariates constant in the model (Table 4).

#### 3.5.3. Sensitivity Analysis Results

Results from the likelihood ratio test showed that the proportionality of odds assumption was not violated (χ^2^ (8) = 13.76; *p* = 0.09). With the exception of age, overall, the results from the multivariable ordinal logistic model were consistent with those from the modified Poisson regression model, confirming the consistency of the predictors at the severe psychological distress level (K6 scores: ≥13; Supplementary Material, Table S1).

## 4. Discussion

In this nationwide study, we found a high overall prevalence of moderate/severe psychological distress (47.2% at K6 scores of ≥5) and several perceived COVID-19-related stressors, including worries and fears of contagion (especially death), serious need for food and money, and concerns about loss of income, or business. The prevalence of suicidal ideation was 1.5%. We also found that those who lived in the Hhohho and Manzini regions, who felt not well informed about COVID-19, who felt lonely, who received COVID-19 food or financial relief from the government, who felt burdened by the lockdown, and who were married, as well as the youth had increased risks of moderate/severe psychological distress during the pandemic.

### 4.1. Psychological Distress and Suicidal Ideation

The prevalence of moderate/severe psychological distress (K6 scores: ≥5) in our study was high, similar to other countries that have been ravaged by the pandemic [11,17,20,57]. Differences in the prevalence in the different studies could be due to different measurements; for example, some used the K10 version of the scale [17,24], and some have either used the K6 as a continuous variable [48] or used the cut-off point of ≥13 to indicate serious mental illness [8,58]. At the K6 cut-off point of ≥13, the 5.4% prevalence is comparable to the 3.4–8% prevalence reported from studies conducted in the U.S. [38,39,40], including during the ongoing pandemic [11]. In addition, our reference period for the symptoms of psychological distress in our study was not “the past 30 days” used in the original K6 which measures point prevalence [37]. Instead, we modified this period to be specific to the pandemic, so that we measured the prevalence of psychological distress during the pandemic (period prevalence), thus trying to exclude pre-pandemic psychological distress symptoms. Kessler et al. [40] provided guidance on the recall periods for the K6 as used in the WHO World Mental Health survey initiative, stating: “… the decision about which recall period was used hinged on whether the investigators were interested in calibrating SMI point prevalence (most useful for screening in clinical settings), 12-month prevalence (most useful for estimating prevalence in surveys used for health-policy planning purposes, as the year is the usual health-policy planning period), or both”. Thus, since our study was population-based, the period prevalence reference period was more appropriate.

Other reasons for the discrepancies in the prevalence could be due to country contexts (e.g., countrys’ response to the pandemic, availability of mental health services, etc.), the period of data collection, as well as the stage of the pandemic during the different data collection periods. However, considering the short coverage period for our study, it is expected that the prevalence rose throughout the pandemic, as seen in other countries [11,59,60].

Also worth noting is that even though the K6 demonstrated a good and acceptable Cronbach’s alpha of 0.79 in our study, it was lower than those reported during its development (0.89 in a telephone pilot sample) [37] and in subsequent studies (ranging between 0.96 and 0.97) [40]. The potential reasons for the lower reliability in our study could be that the instrument was originally developed and validated in native English speakers, whereas for our sample, English was their second language. The K6 website (https://www.hcp.med.harvard.edu/ncs/k6_scales.php (accessed on 2 May 2020)) also does not yet have a SiSwati version of the instrument, which we could have otherwise used. Even though the reported Cronbach’s alpha of the K6 was acceptable, this statistic is not an adequate measure of instrument reliability [61], and therefore, it does not substitute the need for specific validation studies of the K6 in the studied population. Third, as already mentioned, we measured period prevalence (since the pandemic started), whereas the original version assesses point prevalence (last 30 days), which could have also impacted the performance of the scale in our study. However, despite the aforementioned discrepancies and limitations of using the K6 in our study, we do not believe that our results were adversely affected by its lower reliability than previously reported ones, since it still demonstrated good internal consistency in this study.

Our choice of using the sub-threshold cut-off point (K6 scores: ≥5) is supported by the literature [11,41,42,43]. Prochaska et al. [39] argued that due to the low sensitivity of the K6 when used at the K6 cut-off points of ≥13, especially at the population level, it results in some respondents with significant mental distress going undetected. Lee, et al. [62] and Sakurai et al. [63] suggested that different “optimal” cut-offs may be used with the K6, depending on the prevalence in the target population, cost-effectiveness, and purpose of the screening, e.g., screening individuals vs. generating prevalence estimates in an epidemiological survey where the cut-off can be selected at a level that can also capture subthreshold cases. Prochaska et al. [39] found that 27.9% of respondents in their study identified themselves as experiencing moderate mental distress (5 ≤ K6 scores < 13) that impacted functioning across multiple impairment domains (work, household, social, family/friends, and disability) and was associated with increased utilization of mental health treatment. Using the Youden index and the “shortest distance to upper left corner” methods—the most suitable methods to determine an optimal cut-off point, because they are least dependent on population prevalence. In a community and hospital-based Japanese sample, Sakurai et al. [63] found an optimal cut-off point of 4/5 for the K6. Thus, the authors [63] recommended that a cut-off point of 4/5 for the K6 may be used in the screening of mood/anxiety disorders in the general population as well as in epidemiological studies on depression or psychological distress, whereas the 12/13 cut-off point may be used if a screening program targets severe mental illness, has limited resources for secondary screening (e.g., manpower of health care professionals), and thus expects a high post probability of the disorders in the positives. Kessler et al. [37] also pointed out that dimensional measures of nonspecific psychological distress like the K6 distinguish community cases based on severity rather than purely on diagnosis. For that reason, in this study, the K6 was used to assess the frequency and severity of psychological distress symptoms for policy-planning purposes rather than for clinical diagnostic purposes. In any case, performing weighted statistical multivariate modelling at the K6 cut-off point of ≥13 would not have been appropriate in our case due to the few observations in this category.

In Eswatini, which is a culturally conservative society, individuals’ and family’s mental health status may have been uniquely and negatively affected by the measures for containing the pandemic. For example, traditional burial rituals, e.g., washing corpses, prolonged mourning (known as kufukama), were banned during the lockdown to reduce the risk of transmission. As the country’s mortuaries became full, the government instructed families to bury relatives within three days compared to the one-to-four weeks mourning periods before the pandemic; night vigils were also banned, and all burials were held within two hours, giving people less time to mourn. Therefore, coupled with school closures, banning of any social or religious gatherings, loss of jobs, and income for a large sector may have potentially induced the observed moderate/severe psychological distress among the populace.

While it may be reasonably expected that a country like Eswatini, which previously dealt with infectious diseases such as HIV and Tuberculosis (TB), would have relatively greater availability of infectious disease services than other African countries due to its high burden of HIV or TB programs that may not be the case due to several reasons. First, before COVID-19, Eswatini had never experienced an acute and rapidly spreading infectious disease, unlike some countries in West Africa (e.g., with Ebola); hence, there was no prior in-country experience to draw from. Second, the COVID-19 mitigation in Eswatini was not placed under existing infectious disease programs. Instead, it was placed under the country’s Natural Disaster Management Agency (NDMA). Unfortunately, this resulted in poor coordination between the already existing health programs that had experience in handling chronic infectious pandemics like HIV and TB. Third, the country’s NMDA had never handled any health-related disaster before, and they were not only ill-equipped with regards to financial and physical resources to deal with a pandemic of this nature, but also lacked the technical expertise to do so. Therefore, experience gained from existing infectious disease programs was not fully harnessed.

The suicidal ideation prevalence in our study was lower than 6.1% reported in New Zealand [17]. A study conducted in 2014 in Eswatini found a 9.3% prevalence of suicidal ideation over 12 months among a nationally representative sample of adults (15–69 years) [29]. Differences in the prevalence from the two studies could be that our assessment was in reference to the first three months of the pandemic, and our question on suicidal ideation was specific to the pandemic. Hence, our estimate was lower than reported from the study conducted in 2014. However, considering the short coverage period for our study, the 1.5% prevalence can be considered to be high, and it is anticipated that it rose during the pandemic, as observed in other contexts. For example, in the U.S., a collection of three-month cross-sectional data during the beginning of the pandemic revealed that the percentage of suicidal ideation was increased each month for those under the lockdown [64]. In the Czech Republic, the prevalence of suicide risk tripled from 3.9% in 2017 to 11.9% in 2020 [60]. However, a recent systematic review on suicidal outcomes during major international respiratory outbreaks, including COVID-19, found weak support for an association between pandemics, suicide, and suicide-related outcomes [65], underscoring the need for longitudinal studies.

### 4.2. COVID-19-Related Stressors

The findings of fear of contracting contagion, loneliness, disturbed sleep, food insecurity, and financial worries are not unexpected based on results from other studies [13,21,66]. In Australia, Newby et al. [59] found that 25.9% of their participants were very or extremely worried about contracting COVID-19, 52.7% were worried about their family and friends contracting COVID-19, and 50% were concerned about uncertainty, loneliness, and financial worries. In the Czech Republic, Winkler et al. [60] found that strong worries about the health and/or economic consequences of COVID-19 were associated with increased odds of having a mental disorder. The finding on disturbed sleep due to worries was not surprising judged from the new realities that came with the pandemic, including perceived and real threats posed by the uncertainty of the situation, e.g., uncertainties regarding a cure, fear of the unknown, and fear of contracting and dying from the disease, especially at the time of data collection for this study. These findings may be explained by the fact that in a pandemic fear is common due to reactions of individuals’ adaptive defense mechanisms for survival via several biological processes in preparation for a response to the potentially threatening event, which however increases anxiety and stress levels in healthy individuals, especially when it is chronic or disproportionate [67]. Financial worries and food insecurities are also expected during a lockdown, as people lose jobs or means of business, shops close and movements are restricted. A recent qualitative study found that people living in informal settlements in South Africa were affected by lack of savings, loss of income, shortage of food and hunger, anxiety, and depression during the pandemic [68].

### 4.3. Predictors of Psychological Distress

We found that the youth (18–24 years) had a significantly increased risk of moderate/severe psychological distress than those aged 25–59 years, similar to findings from other studies [10,11,21], possibly because a majority of the people in this age group were still at school but schools were closed at the beginning of the pandemic which might have increased their worries about school closures. Secondly, this group includes adolescents (18–19 years) who usually place value in social interactions with their school peers or sports mates, but both of these activities were abruptly suspended during the lockdown, which might induce some psychological distress among the youth. Thirdly, some of the people in this subgroup may be looking for jobs or maybe under temporary or short contract employment, they might be worried about job security during the lockdown, unlike older adults who might already have stable jobs and therefore might have continued working at home.

In this study, being married was also found to be associated with a higher risk of moderate/severe psychological distress, similar to findings from previous studies [11,24]. This may be due to the lockdown, as some couples found themselves “locked” under one roof for prolonged periods than under normal circumstances, which might have sparked conflicts. Other potential sources of distress among this group, especially those with children, may include childcare, homeschooling [11], increased utility bills, as families spend extended time indoors, food insecurity, etc. However, other studies have found that unmarried individuals reported higher psychological distress during the pandemic [8,48,58], calling for studies that will be statistically powered to specifically examine this association during the pandemic. We also found an increased risk of moderate/severe psychological distress among those who felt lonely, similar to other studies which found that loneliness was associated with higher levels of mental health symptomatology during the COVID-19 pandemic [66].

It was not surprising that participants living in the Hhohho and Manzini regions had an increased risk of moderate/severe psychological distress, since these are the regions that have been severely affected by the pandemic [3]. In fact, during the first wave, the City of Manzini and surrounding areas located in the Manzini region became the epicenter, whereas Mbabane (the capital city) and surrounding areas located in the Hhohho region became the epicenter during the second wave. These regions, being home to the country’s two major cities and with Manzini housing the largest industrial town, were the most affected by lockdowns, since most of the businesses are located there and most of the employed population resides. Hence, participants from those areas might be at an increased risk of moderate/severe psychological distress. That could also explain the finding that those who felt burdened by the lockdown had an increased risk of moderate/severe psychological distress since the lockdowns were mainly enforced in these areas more than anywhere else, e.g., through police patrols and roadblocks.

We also found an increased risk of moderate/severe psychological distress among those who felt not well informed about COVID-19 than those who felt well informed. However, current evidence regarding this association is mixed. For example, Jungmann and Witthöft [49] found an inverse correlation between feelings about the pandemic and anxiety, whereas Lei, et al. [69] found increased anxiety among participants who reported a higher self-evaluated level of knowledge about the virus. On the contrary, Wang et al. [21] found that having specific up-to-date and accurate health information about the outbreak was associated with lower levels of stress, anxiety, and depression. The mixed results call for objective measurement of knowledge in order to examine this finding further.

This study also revealed that participants whose households received COVID-19 food or financial relief from the government had an increased risk of moderate/severe psychological distress. Although not overtly intuitive, this finding is not unexpected since for households to receive such relief in the first place. They must be of a low socioeconomic status, e.g., having no stable income or no breadwinner, and low socioeconomic status was linked to psychological distress [23]. In the U.S., unemployed men and women receiving means-tested or welfare benefits were found to be more likely to report depression, in both the short and long term [70]. In another U.S. study, depression and anxiety symptoms increased among households who reported receiving unemployment insurance benefits, relative to a period when an unemployment insurance benefit was in effect [71]. Therefore, the direction of the association for this finding may not necessarily mean that receiving the COVID-19 relief materials induced moderate/severe psychological distress among recipients, but rather that recipients may have already been moderately/severely psychologically distressed (reverse causation) due to their difficult financial or social conditions. Thus, longitudinal studies are warranted to establish the temporality between these variables which may, in turn, serve as an evaluation of the effectiveness of the COVID-19 relief program in reducing the psychological burden induced by the pandemic among the recipients. 

It is important to note that the predictors of psychological distress were comparable in both the multivariable modified Poisson and ordinal logistic regression models, implying that the choice of the K6 cut-off point did not influence significantly what we eventually reported. In our case, the purpose of the ordinal logistic regression analysis was to assess if the predictors were the same at the K6 cut-off points of ≥5 and ≥13. We could have fitted a multinomial regression model, similar to previous studies [39]; however, in our sample, the group aged at ≥13 was not adequate to fit a multivariable multinomial logistic model. Since the K6 cut-off points have some order or ranking, the alternative was to fit an ordinal logistic regression model, a case in which the odds ratio represents the odds of the higher category as compared to all lower categories combined. Our results showed that the predictors of psychological distress were similar at different levels or categorizations of the K6 scores.

### 4.4. Strengths and Limitations

To our knowledge, this is the first published study that utilized telephones to collect research data in Eswatini, and it is the first published study investigating the psychological status of the general public during the COVID-19 pandemic in Eswatini. Therefore, our methodology will inform future researchers of the feasibility of telephone-based research in Eswatini. These baseline findings will inform ongoing and future national mitigation efforts aimed at reducing psychological problems induced by the COVID-19 pandemic, as well as by future outbreaks. With COVID-19 being a novel disease, this study adds to the growing body of empirical evidence regarding the impact of the pandemic on mental health. We used an instrument with a well-established cross-cultural validity to measure our main outcome variable [40,45]. To the best of our knowledge, this is the first published study that used the K6 in Eswatini, which on its own contributes to the literature regarding the internal consistency of the K6 and will, therefore, inform future studies conducted in this population. However, we did not assess the validity of the original K6 and the translated version used in this study; thus, studies assessing its validity in this population are warranted. Ali et al. [45] noted that cross-cultural application of a screening tool requires that its validity is assessed against gold-standard diagnostic interviews, e.g., fully structured research diagnostic interviews administered by lay interviewers [40].

Despite the aforementioned strengths, the study still has several limitations. First, in the absence of comparable studies on the psychological distress status of the population before the pandemic in Eswatini, we cannot be certain that all the prevalence values of psychological distress observed in our sample were due to the pandemic, especially because other events and changes occurred before and continued to occur during the pandemic. It should also be noted that the data were collected in the early stages of the pandemic and therefore may not reflect the situation during the entire pandemic. Second, coverage survey bias (which was high in our case since the landline service covers approximately 4.5% of the country’s population) and selection bias are inherent in telephone-based surveys, as they exclude those members of the population who do not have telephone lines, of which unweighted findings are not generalizable to the entire Eswatini adult population. In addition, the respondents were from households with telephone landlines and only EPTC subscribers, which underrepresents lower-income households. However, we tried to minimize coverage bias by using PPS during sampling, and we applied post-stratified sample weights during analysis, of which the weighted results are generalizable to the general adult (15+ years) population in Eswatini. In addition, we collected the data at a time when the measures of the partial lockdown were strictly enforced by the government and many emplooyees were working from home, whom we would have not enrolled had the data been collected at another time e.g. when the lockdown had been lifted or was not in place.

Third, telephone surveys can only serve to obtain baseline information on the studied variables but do not explore them in-depth. Fourth, we did not collect specific information on all known predictors of psychological distress, such as history of mental problems or presence of specific chronic illnesses (e.g., hypertension, diabetes, or HIV status), but these could increase people’s concerns during the pandemic. Fifth, using only one item to measure suicidal ideation does not fully capture the concept, which may result in a low level of ideation reporting in our study, hence creating the aforementioned statistical issues related to this outcome. Lastly, self-reporting of behaviors may not be the same as actual behaviors, of which some reporting bias may be present; however, this is assumed to occur at random.

## 5. Conclusions

Our study revealed that the prevalence of moderate/severe psychological distress in the general population was high and that fear of contracting the contagion, loneliness, disturbed sleep, food insecurity, and financial worries were not uncommon during the COVID-19 pandemic. We also found that the general Eswatini adult population who lived in the Hhohho and Manzini regions, who felt burdened by the lockdown, who felt not well informed about COVID-19, who felt lonely, who aged at 18–24 years, who were married, and those who received COVID-19 food or financial relief from the government had increased risks of moderate/severe psychological distress during the early stages of the pandemic. Psychological interventions should be implemented urgently, especially for those subgroups that were found to have an increased risk of moderate/severe psychological distress in this study. These include decentralizing mental health services to communities through the existing community outreach program to provide counseling to affected individuals as well as referring those who require material support to the relevant existing structures to reduce their stressors. Mental health service providers should also engage communities to ascertain the kind of material support they need to ensure that initiatives respond directly to needs of disadvantaged groups during the ongoing pandemic. 

There is a need to repeat the survey, once the lockdown is lifted to assess changes in the mental health status over the COVID-19 pandemic. Qualitative studies are also warranted to investigate the reasons for the observed psychological distress prevalence during the pandemic and the reasons for the suicidal thoughts reported by the participants. Future studies may also consider measuring suicidal behavior as a whole (suicidality) using validated scales. Lastly, there is a need for studies specifically powered to validate the translated version of the K6 and its translated version in the studied population.

## Figures and Tables

**Table 1:** Participants’ background characteristics (unweighted *N* = 993; weighted *N* = 700,051).

Characteristic	Unweighted *n*	Unweighted (%)	Weighted (%; 95% CI)
Age in years			
18–24	233	23.5	32.4 (32.4, 32.4)
25–59	587	59.1	48.7 (48.7, 48.7)
60–92	173	17.4	18.9 (18.9, 18.90
Sex			
Female	718	72.3	52.3 (52.3, 52.3)
Male	275	27.7	47.7 (47.7, 47.7)
Marital status			
Single	448	45.1	49.5 (46.4, 52.6)
Married/cohabiting	478	48.1	45.8 (42.5, 49.1)
Widowed/divorced/separated	67	6.8	4.7 (3.3, 6.7)
Religion			
Christian	975	98.2	97.0 (95.0, 98.3)
Other/atheist	18	1.8	3.0 (1.7, 5.0)
Highest education level attended			
Never schooled	24	2.4	2.9 (1.8, 4.8)
Primary/Sebenta ^a^	65	6.6	7.6 (5.7, 10.0)
Secondary	130	13.1	13.6 (11.0, 16.7)
High school	349	35.2	39.3 (35.5, 43.3)
Tertiary	425	42.8	36.6 (32.9, 40.5)
Residential area			
Rural	400	40.3	73.1 (73.1, 73.1)
Urban	593	59.7	26.9 (26.9, 26.9)
Subjective socioeconomic status			
Very poor/poor	92	9.3	9.6 (7.5, 12.2)
Middle	793	79.9	80.0 (76.5, 83.1)
Very rich/rich	30	3.0	2.9 (1.7, 4.7)
Can’t tell	78	7.9	7.6 (5.7, 10.0)
Region			
Hhohho	302	30.4	29.8 (29.8, 29.8)
Manzini	334	33.6	33.8 (33.8, 33.8)
Shiselweni	173	17.4	17.7 (17.7, 17.7)
Lubombo	184	18.5	18.7 (18.7, 18.7)
Self/family member lost job or business means due to pandemic			
Yes	352	35.5	36.2 (32.3, 40.2)
No	641	64.6	63.8 (59.8, 67.7)
Know people diagnosed with COVID-19			
Yes	68	6.9	4.8 (3.4, 6.7)
No	925	93.2	95.2 (93.3, 96.6)
Received food/financial relief from the government during the lockdown			
Yes	99	10.0	11.4 (9.0, 14.4)
No/not sure	894	90.0	88.6 (85.6, 91.0)
Feel well informed about COVID-19			
Yes	642	64.7	64.9 (60.9, 68.7)
No	131	13.2	14.5 (11.8, 17.7)
Not sure	220	22.2	20.6 (17.6, 24.0)
Perceived ability to avoid contracting coronavirus			
Very easy/easy	382	38.5	40.0 (36.1, 44.2)
Moderate	158	15.9	15.9 (13.1, 19.1)
Very difficult/difficult	453	45.6	44.1 (40.0, 48.2)
Perceived probability of contracting coronavirus			
Very low/low	317	31.9	31.2 (27.5, 35.1)
Moderate	194	19.5	19.3 (16.3, 22.7)
Very high/high	462	46.5	47.8 (43.8, 51.9)
Not sure	20	2.0	1.7 (0.9, 3.1)
Perceived severity if sick with COVID-19			
Not severe/less severe	313	31.5	31.7 (28.1, 35.6)
Moderate	236	23.8	21.7 (18.5, 25.2)
Very severe/severe	313	31.5	33.4 (29.8, 37.3)
Not sure	131	13.2	13.2 (10.7, 16.3)
Prevalence of suicidal ideation			
Had suicidal thoughts	17	1.7	1.5 (0.8, 2.7)
No suicidal thoughts	976	98.3	98.5 (97.3, 99.2)
Prevalence of psychological distress			
None/low (K6 0–4)	499	50.3	52.8 (48.7, 56.9)
Moderate (K6 5–12)	415	41.8	41.7 (37.7, 45.8)
Severe (K6 ≥13)	79	8.0	5.4 (3.9, 7.5)

Note: ^a^ Sebenta is a form of informal education targeting “old-age learners”, offering lessons equivalent to grades 1–7 under the formal education system; K6, Kessler 6 scale; CI, confidence interval.

**Table 2:** Weighted distribution of background characteristics of the sample by suicidal ideation (*N* = 993).

Background Characteristic	No Suicidal Thoughts *n* (Weighted Percentage (%))	Having Suicidal Thoughts *n* (Weighted Percentage (%))
Age in years		
18–24	228 (32.4)	5 (33.8)
25–59	576 (48.6)	11 (56.9)
60–92	172 (19.0)	1 (9.4)
Sex		
Male	271 (47.9)	4 (32.7)
Female	705 (52.1)	13 (67.3)
Marital status		
Single	437 (49.1)	11 (74.7)
Married/cohabiting	473 (46.1)	5 (25.3)
Widowed/divorced/separated	66 (4.8)	1 (0.001)
Highest educational level attended		
Never schooled	24 (3.0)	0 (0.0)
Primary/Sebenta ^a^	65 (7.7)	0 (0.0)
Secondary	128 (13.7)	2 (6.9)
High school	342 (39.2)	7 (47.4)
Tertiary education	417 (36.5)	8 (45.7)
Area of residence		
Urban	582 (26.8)	11 (39.5)
Rural	394 (73.3)	6 (60.6)
Subjective socioeconomic status		
Very poor/poor	91 (9.5)	1 (11.2)
Middle	782 (80.1)	11 (74.5)
Very rich/rich	29 (28.9)	1 (0.001)
Can’t tell	74 (7.5)	4 (14.3)
Region of residence		
Hhohho	295 (29.5)	7 (49.8)
Lubombo	183 (18.8)	1 (11.4)
Manzini	330 (34.1)	4 (18.1)
Shiselweni	168 (17.6)	5 (20.8)
Feel well informed about COVID-19		
Yes	628 (64.6)	14 (87.8)
No	130 (14.6)	1 (5.2)
Not sure	218 (20.8)	2 (6.9)
Perceived ability to avoid contracting COVID-19		
Very easy/easy	374 (39.8)	8 (54.1)
Moderate	156 (16.0)	2 (7.6)
Very difficult/difficult	446 (44.2)	7 (38.3)
Perceived probability of contracting COVID-19		
Very low/low	315 (31.5)	2 (11.4)
Moderate/	189 (19.1)	5 (31.4)
Very high/high	453 (47.8)	9 (50.3)
Not sure	19 (1.6)	1 (6.9)
Perceived severity if sick with COVID-19		
Not severe/less severe	307 (31.7)	6 (32.9)
Moderate	234 (21.9)	2 (6.8)
Very severe/severe	309 (33.6)	4 (22.9)
Not sure	126 (12.9)	5 (37.4)
Self/family member lost job/business due to pandemic		
Yes	348 (36.2)	4 (32.1)
No	628 (63.8)	13 (67.9)
Knows people diagnosed with COVID-19 *		
Yes	64 (4.7)	4 (9.7)
No	912 (95.3)	13 (90.3)
Burdened by the lockdown itself		
Yes	210 (20.8)	2 (17.2)
No	766 (79.2)	15 (82.8)
Received COVID-19 food/financial relief from the government during lockdown		
Yes	98 (11.6)	1 (0.7)
No/Not sure	878 (88.4)	16 (99.3)
Felt lonely during the lockdown		
Never/rarely	747 (78.4)	13 (87.1)
Sometimes/Most of the time/always	229 (21.6)	4 (12.9)
Psychological distress		
None/low (K6 scores: 0–4)	489 (52.7)	10 (63.0)
Moderate/severe (K6 scores: ≥5)	487 (47.3)	7 (37.0)

Notes. ^a^ Sebenta is a form of informal education targeting ‘old-age learners’, offering lessons equivalent to grades 1–7 under the formal education system. All “*n*” are unweighted; not all percentages add up to 100% due to rounding; * *p*-value of unweighted Fisher exact’s test, 0.02; K6, Kessler 6 scale.

**Table 3:** COVID-19-related stressors among adults in Eswatini (unweighted *N* = 993, weighted *N* = 700,051).

Variable	Unweighted *n*	Unweighted Percentage (%)	Weighted Percentage (%; 95% CI)
Afraid of COVID-19			
Yes	235	23.7	23.9 (20.6, 27.6)
No	739	74.4	74.3 (70.6, 77.8)
Not sure	19	1.9	1.8 (0.9, 3.5)
Felt burdened by the lockdown itself	212	21.4	20.8 (17.7, 24.3)
Worried about risk of contracting COVID-19	141	14.2	15.0 (12.2, 18.4)
Experienced at least one form of abuse during the lockdown ^a^	41	4.1	4.4 (3.0, 6.6)
Sometimes/most of the time/always felt lonely during lockdown	233	23.5	21.4 (18.2, 25.1)
Sometimes/most of the time/always worried about the pandemic such that can’t sleep at night	237	23.9	22.4 (19.1, 26.0)
Fears about ^b^:			
being separated from family	254	25.6	25.2 (21.8, 28.8)
being hospitalized due to COVID-19	222	22.4	22.0 (18.8, 25.5)
dying from COVID-19	505	50.9	53.1 (49.0, 57.1)
Seriously in need of ^b^:			
money during the lockdown	198	19.9	19.8 (16.7, 23.3)
medication during the lockdown	65	6.6	6.4 (4.7, 8.5)
food during the lockdown	574	57.8	58.4 (54.4, 62.2)
Concerned about ^b^:			
loss of income/job/business	171	17.2	15.7 (13.0, 18.9)
difficulty to keep away from crowds	48	4.8	5.1 (3.6, 7.3)
nonavailability of transport	37	3.7	3.5 (2.3, 5.3)
misinformation/fake news	36	3.6	2.6 (1.7, 4.2)
inability to pay rent	25	2.5	3.1 (1.9, 5.0)

Note: not all percentages add up to 100% due to rounding; ^a^ including verbal, emotional, physical, and sexual abuse; ^b^ Each variable was asked separately, and hence, total column percentages do not need to add up to 100%.

**Table 4:** Weighted modified Poisson regression models depicting predictors of moderate/severe psychological distress among adults during the COVID-19 pandemic in Eswatini (*N* = 993).

Variable	None/Low Distress *n* (wt Percentage (%) ^a^)	Moderate/Severe Distress *n* (wt Percentage (%) ^a^)	CRR (95% CI)	ARR (95% CI)
Age in years (ref: 25–59)				
18–24	111 (30.1)	122 (35.0)	1.11 (0.92, 1.35)	1.34 (1.05, 1.70) *
60–92	98 (20.1)	75 (17.6)	0.96 (0.74, 1.23)	0.96 (0.76, 1.21)
Male (ref: Female)	158 (50.8)	117 (44.2)	0.87 (0.73, 1.04)	0.97 (0.82, 1.15)
Marital status (ref: Single)				
Married/cohabiting	248 (46.4)	230 (45.1)	1.00 (0.83, 1.20)	1.37 (1.09, 1.72) **
Widowed/divorced/separated	29 (3.5)	38 (6.1)	1.31 (0.94, 1.81)	1.28 (0.94, 1.76)
Highest educational level attended (ref: Tertiary)				
Never schooled	14 (3.4)	10 (2.5)	1.00 (0.52, 1.91)	-
Primary/Sebenta	33 (8.8)	32 (6.2)	0.98 (0.65, 1.48)	-
Secondary	53 (9.6)	77 (18.0)	1.58 (1.24, 2.02) ***	-
High school	164 (36.3)	185 (42.6)	1.29 (1.05, 1.60) *	-
Urban (ref: Rural)	277 (25.0)	316 (29.1)	1.11 (0.94, 1.32)	
Subjective socioeconomic status (ref: Can’t tell)				
Very poor/poor	36 (8.2)	56 (11.1)	1.29 (0.84, 1.97)	-
Middle	406 (80.0)	387 (80.0)	1.11 (0.77, 1.60)	-
Very rich/rich	17 (3.5)	13 (2.1)	0.82 (0.38, 1.75)	-
Region (ref: Lubombo)				
Hhohho	119 (23.8)	183 (36.7)	1.76 (1.31, 2.36) ***	1.63 (1.24, 2.15) **
Manzini	161 (33.3)	173 (34.4)	1.45 (1.07, 1.97) *	1.42 (1.07, 1.89) *
Shiselweni	102 (19.2)	71 (15.9)	1.29 (0.91, 1.81)	1.28 (0.93, 1.77)
Feel well informed about COVID-19 (ref: Yes)				
No	40 (8.6)	91 (21.1)	1.62 (1.35, 1.95) ***	1.59 (1.32, 1.91) ***
Not sure	102 (20.5)	118 (20.7)	1.12 (0.90, 1.40)	1.05 (0.86, 1.29)
Perceived ability to avoid contracting COVID-19 (ref: Moderate)				
Very easy/easy	220 (45.6)	162 (33.8)	0.99 (0.74, 1.34)	-
Very difficult/difficult	189 (36.4)	264 (52.7)	1.41 (1.07, 1.85) *	-
Perceived probability of contracting COVID-19 (ref: Very low/low)				
Moderate/	94 (17.3)	100 (21.5)	1.27 (0.99, 1.63)	-
Very high/high	224 (46.0)	238 (49.8)	1.19 (0.96, 1.47)	-
Not sure	9 (2.1)	11 (1.2)	0.81 (0.37, 1.79)	-
Perceived severity if sick with COVID-19 (ref: Not severe/less severe)				
Moderate	127 (23.9)	109 (19.2)	1.00 (0.77, 1.31)	-
Very severe/severe	133 (27.6)	180 (39.9)	1.35 (1.08, 1.67) **	-
Not sure	66 (13.7)	65 (12.7)	1.08 (0.80, 1.47)	-
Self/family member lost job/business due to the pandemic (Ref: No)	149 (32.9)	203 (39.8)	1.17 (0.98, 1.39)	-
Knows people diagnosed with COVID-19 (ref: No)	30 (4.0)	38 (5.6)	1.18 (0.85, 1.64)	-
Burdened by the lockdown itself (ref: Not)	86 (16.1)	126 (26.0)	1.34 (1.11, 1.61) **	1.33 (1.11, 1.59) **
Received COVID-19 food/financial relief from the government during the lockdown (ref: No/not sure)	38 (8.7)	61 (14.5)	1.31 (1.05, 1.64) *	1.35 (1.10, 1.65) **
Sometimes/most of the time/always felt lonely during the lockdown (ref: Never/rarely felt lonely)	41 (8.6)	192 (35.8)	2.05 (1.75, 2.38) ***	2.01 (1.23, 2.34) ***

Notes. * *p* < 0.05; ** *p* < 0.01; *** *p* < 0.001; CRR, crude risk ratio; ARR, adjusted risk ratio; CI, confidence interval; ref, reference category; wt, weighted; ^a^ Column totals.

## Data Availability

The research data are accessible from the first author upon reasonable request. Otherwise, all supporting information is provided in the article.

## Supplementary Materials

The following are available online at https://www.mdpi.com/article/10.3390/ijerph18136700/s1, Table S1: Weighted ordinal logistic regression models depicting predictors of psychological distress among adults during the COVID-19 pandemic in Eswatini.
