# Supplementary material for: Suicidal Ideation and Predictors of Psychological Distress during the COVID-19 Pandemic in Eswatini: A Population-Based Household Telephone Survey
Source: Int J Environ Res Public Health. 2021 Jun 22;18(13):6700. doi: 10.3390/ijerph18136700 (PMC8296988; doi:10.3390/ijerph18136700)
Supplement: Supplementary file 1 [file ijerph-18-06700-s001.zip › ijerph-1243353-supplementary.pdf]

Table S1. Weighted ordinal logistic regression models depicting predictors of psychological distress among adults during the COVID-19 pandemic in Eswatini ($N = 993$)

Variable	Psychological distress Level			COR (95% CI)	AOR (95% CI)
	None/low ($n = 499$) *n* (wt[a] %)	Moderate ($n = 415$) *n* (wt[a] %)	Severe ($n = 79$) *n* (wt[a] %)		
Age in years (ref: 25-59)					
18-24	111 (30.1)	108 (36.1)	14 (27.3)	1.16 (0.80, 1.68)	1.59 (0.09, 2.75)
60-92	98 (20.1)	63 (18.0)	12 (14.0)	0.89 (0.57, 1.38)	0.84 (0.51, 1.37)
Male (ref: Female)	158 (50.8)	109 (46.6)	8 (26.3)	0.72 (0.52, 1.00)	0.83 (0.58, 1.18)
Marital status (ref: Single)					
Married/cohabiting	248 (46.4)	195 (45.2)	35 (44.6)	0.99 (0.71, 1.39)	1.84 (1.11, 3.05)*
Widowed/divorced/separated	29 (3.5)	30 (6.2)	8 (5.3)	1.67 (0.83, 3.37)	1.50 (0.67, 3.39)
Highest educational level attended (ref: Tertiary)					
Never schooled	14 (3.4)	9 (2.4)	1 (3.1)	1.05 (0.36, 3.07)	-
Primary/*Sebenta*	33 (8.8)	27 (6.4)	5 (4.5)	0.97 (0.50, 1.86)	-
Secondary	53 (9.6)	65 (18.7)	12 (12.7)	2.35 (1.46, 3.80)***	-
High school	164 (36.3)	149 (40.6)	36 (58.5)	1.69 (1.16, 2.45)**	-
Urban (ref: Rural)	277 (25.0)	260 (29.4)	56 (26.9)	1.21 (0.88, 1.65)	
Subjective socioeconomic status (ref: Can't tell)					
Very poor/poor	36 (8.2)	46 (10.3)	10 (17.0)	1.57 (0.68, 3.65)	-
Middle	406 (80.0)	333 (81.4)	54 (69.0)	1.09 (0.56, 2.13)	-
Very rich/rich	17 (3.5)	9 (2.3)	4 (0.3)	0.63 (0.20, 2.03)	-
Region (ref: Lubombo)					
Hhohho	119 (23.8)	149 (36.6)	34 (36.8)	2.69 (1.65, 4.38)***	2.74 (1.60, 4.68)***
Manzini	161 (33.3)	148 (34.2)	25 (35.9)	1.85 (1.14, 3.03)*	2.00 (1.17, 3.42)*
Shiselweni	102 (19.2)	63 (16.2)	8 (13.2)	1.47 (0.85, 2.53)	1.47 (0.81, 2.67)
Feel well informed about COVID-19 (ref: Yes)					
No	40 (8.6)	75 (20.6)	16 (24.5)	2.80 (1.79, 4.38)***	3.01(1.84, 4.92)***
Not sure	102 (20.5)	95 (20.1)	23 (25.2)	1.26 (0.84, 1.90)	1.14 (0.73, 1.78)
Perceived ability to avoid contracting COVID-19 (ref: Moderate)					
Very easy/easy	220 (45.6)	140 (33.8)	22 (33.9)	1.02 (0.63, 1.65)	-
Very difficult/difficult	189 (36.4)	217 (52.0)	47 (58.1)	1.96 (1.24, 3.11)**	-
Perceived probability of contracting COVID-19 (ref: Very low/low)					
Moderate/	94 (17.3)	83 (21.1)	17 (24.7)	1.64 (1.03, 2.60)*	-
Very high/high	224 (46.0)	193 (48.7)	45 (58.7)	1.43 (0.98, 2.08)	-

Variable	Psychological distress Level			COR (95% CI)	AOR (95% CI)
	None/low (n = 499) n (wt[a] %)	Moderate (n = 415) n (wt[a] %)	Severe (n = 79) n (wt[a] %)		
Not sure	9 (2.1)	9 (0.9)	2 (3.3)	0.82 (0.21, 3.30)	-
Perceived severity if sick with COVID-19 (ref: Not severe/less severe)					
Moderate	127 (23.9)	99 (19.6)	10 (16.5)	1.00 (0.64, 1.58)	-
Very severe/severe	133 (27.6)	140 (39.0)	40 (46.8)	1.81 (1.21, 2.71)**	-
Not sure	66 (13.7)	56 (12.5)	9 (14.4)	1.18 (0.68, 2.04)	-
Self/family member lost job/business due to pandemic (Ref: No)	149 (32.9)	158 (37.5)	45 (57.9)	1.45 (1.03, 2.03)*	-
Knows people diagnosed with COVID-19 (ref: No)	30 (4.0)	30 (5.0)	8 (9.6)	1.52 (0.72, 3.18)	-
Burdened by the lockdown itself (ref: Not)	86 (16.1)	111 (27.3)	15 (16.0)	1.64 (1.14, 2.37)**	1.68 (1.12, 2.52)*
Received COVID-19 food/financial relief from the government during lockdown (ref: No/not sure)	38 (8.7)	49 (13.7)	12 (20.4)	1.82 (1.08, 3.07)*	2.06 (1.20, 3.55)**
Sometimes/most of the time/always felt lonely during the lockdown (ref: Never/rarely felt lonely)	41 (8.6)	134 (31.8)	58 (66.7)	6.50 (4.07, 10.36)***	6.83 (4.32, 10.80)***

Notes. *$p < .05$; **$p < .01$; ***$p < .001$; Likelihood ratio test of proportionality of odds: ($\chi 2$ (8) = 13.76, p = .09). COR, crude odds ratio; AOR, adjusted odds ratio; CI, confidence interval; ref, reference category; wt, weighted; [a]Column totals.
